# A Community-based Study of the Willingness to Pay Associated with Screening for Diabetic Retinopathy among Type 2 Diabetes in Kinmen, Taiwan

**DOI:** 10.2188/jea.17.186

**Published:** 2007-12-19

**Authors:** Hui-Chuan Shih, Pesus Chou, Shih-Jen Chen, Jorn-Hon Liu, Fenq-Li Lee, Chi-Ming Liu, Tao-Hsin Tung

**Affiliations:** 1 Department of Nursing, Kaohsiung Armed Forced General Hospital.; 2 Community Medicine Research Center & Institute of Public Health, National Yang-Ming University.; 3 Department of Ophthalmology, Veterans General Hospital.; 4 Cheng Hsin Rehabilitation Medical Center.; 5 Faculty of Medicine, School of Medicine, National Yang-Ming University.; 6 Faculty of Public Health, School of Medicine, Fu-Jen Catholic University.

**Keywords:** Community-based Studies, Diabetic Retinopathy, Diabetes Mellitus, Type 2, Willingness-to-pay, Taiwan

## Abstract

**BACKGROUND:**

In Taiwan, there were few population-based studies of WTP values related to DR screening among persons with type 2 diabetes. This community-based study was to explore the willingness-to-pay (WTP) values for screening for diabetic retinopathy (DR) associated with varying degrees of DR among persons with type 2 diabetes in Kinmen, Taiwan.

**METHODS:**

A total of 725 eligible community-dwelling adults diagnosed with type 2 diabetes received DR screening during 1999-2002 and then evaluated WTP values in 2003. Diagnosis of DR was performed by a panel of ophthalmologists using ophthalmoscopy and a 45-degree color retinal photographs to examine fundus after dilating pupils. WTP values were measured by discrete-choice method.

**RESULTS:**

The 406 adults with type 2 diabetes participating in the WTP survey had a 56% response rate. Of 406 subjects, 265 (65.3%) said they would be willing to pay for DR screening to reduce blindness. The overall mean WTP value was New Taiwan Dollars 468.9±327.7 (US dollars 14.3±10.0). Age was borderline significant (p=0.07) related to WTP values. Those with severe stage DR had higher WTP values for screening than subjects with mild stage.

**CONCLUSIONS:**

Degree of DR was the independent factor affecting WTP values in DR screening among community-dwelling adults with type 2 diabetes.

Development of type 2 diabetes immediately increases a patient's propensity for developing a broad spectrum of irreversible complications. Diabetic retinopathy (DR) is a progressive microangiopathy characterized by small vessel damage and occlusion. In Taiwan, DR is the fourth leading cause of visual impairment, as found in a survey of the suburb Taipei.^1^ Because the average duration of time from development of no diabetic retinopathy (NDR) to blindness is approximately 26.5 years in persons with type 2 diabetes, assessing the progression of DR by screening is a worthwhile preventive measure.^2^

According to welfare economic theory, the benefit to an individual of a service or an intervention is defined as that individual's maximum willingness to pay (WTP) for the service or intervention.^3^ WTP is a contingent valuation and involves using a hypothetical survey to directly ask individuals the maximum amount they are willing to pay for the commodity in question.^4^^,^^5^ Conceptually, for a health improvement, the WTP approach assumes that subject well-being depends on both income and health. If a treatment is introduced that moves health status from a specific disease state to full health, a person's WTP is the maximum amount of money that he/she would pay for treatment that restores to full health while maintaining the same level of overall well-being.^3^

In Taiwan, there were few population-based studies of WTP values related to DR screening among persons with type 2 diabetes. The purpose of this study was to assess WTP values for DR screening associated with varying degrees of DR among community-based adults diagnosed with type 2 diabetes in Kinmen, Taiwan.

## METHODS

### Study Design and Data Selection

**Figure 1.**
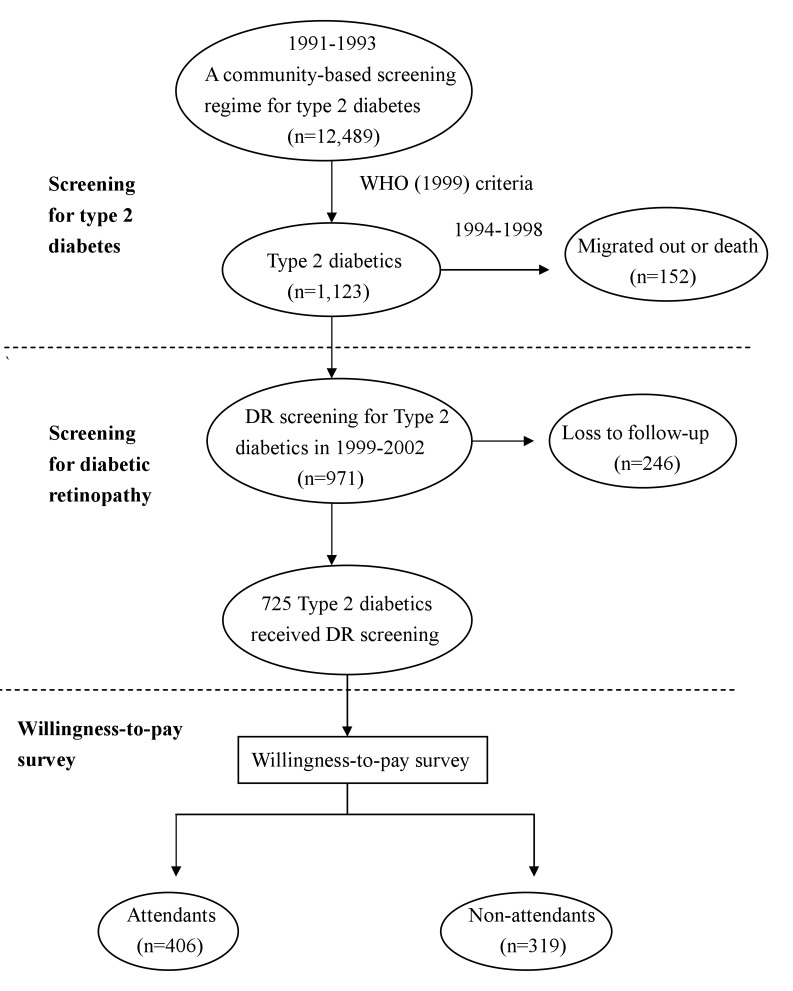
Procedure of willingness-to-pay survey for community-dwelling adults diagnosed with type 2 diabetes in 2003 in Kinmen.

Figure 1 shows the procedures of WTP survey for community-dwelling adults previously diagnosed with type 2 diabetes in this study. Details of the original study design and execution have been described in full elsewhere.^6^ In brief, we conducted a community-based survey for adults diagnosed with type 2 diabetes targeting subjects aged 30 years or older in Kinmen, Taiwan, between January 1991 and December 1993. Another screening program for early detection of DR among adults diagnosed with type 2 diabetes was conducted initially in 1999. Persons selected in the present study was based on the community-based screening for DR among adults diagnosed with type 2 diabetes from 1999 through 2002. The identification for type 2 diabetes was based on the WHO definition in 1985,^7^ that is, subjects with fasting plasma glucose (FPG) ≥140 mg/dL or 2-hr postload ≥200 mg/dL were defined as type 2 diabetes. Subjects with history of type 2 diabetes and received medication were defined as known cases. However, in the ophthalmologic screening done in 1999-2002, the patients that fulfilled the criteria of revised WHO 1999 were enrolled. Additional patients with FPG ≥126 and <139 mg/dL in 1991-1993 were recruited.^8^ Subjects eligible to participate were subsequently asked whether they would be willing to answer questions related to a WTP survey in 2003.

Informed consent was obtained from all participants before the survey was administered. WTP value, related demographic information (sex, age, duration of type 2 diabetes, education, marriage status, and medication) and other personal chronic disease history were collected at one-to-one interviews using a structured questionnaire administered by the Yang-Ming Crusades, organized from the medical students of the National Yang-Ming University. Biological factors (body mass index [BMI], hemoglobin [Hb] A1c, total cholesterol, triglyceride, and urine protein / urine creatinine) were collected from fasting blood and urine samples drawn by public health nurses. Persons unwilling to answer the WTP questions related to type 2 diabetes or DR were excluded from the study.

Access to personal records was approved by the hospital human subjects review board at Cheng-Hsin Rehabilitation Medical Center, Taipei, Taiwan.

### Definition of Diabetic Retinopathy

The diagnosis of DR was based on on-site indirect ophthalmoscopic examination and single-field fundus photographs analyzed later. On-site screening was conducted by two ophthalmologists using indirect ophthalmoscopy after pupil dilatation with topical 0.5% tropicamide. Graders recorded the diagnosis. Then one 45-degree color fundus photograph with Polaroid 600 film (Polaroid, Nieuw-Vennep, Netherlands) was taken per eye, centered at the macula using a Topcon fundus camera (TRC-50VT, Tokyo, Japan). The single-field photographs were printed and graded by two well-trained senior ophthalmologists beginning no later than one month after the screening. Final grading of DR depended on the summed interpretation of the photographs and the recorded indirect ophthalmoscopic gradings. According to the Diabetic Retinopathy Disease Severity Scale,^9^^,^^10^ DR was classified as follows: no diabetic retinopathy (NDR, no abnormalities), mild non-proliferative diabetic retinopathy (mild NPDR, subjects with microaneurysms only), moderate non-proliferative diabetic retinopathy (moderate NPDR, subjects with more than just microaneuysms but less than severe NPDR), severe non-proliferative diabetic retinopathy (severe NPDR, subjects with any of the following: more than 20 intraretinal hemorrhages in each of 4 quadrants; definite venous beading in 2+ quadrants; prominent intraretinal microvascular abnormalities in 1+ quadrant; and no signs of proliferative diabetic retinopathy), and proliferative diabetic retinopathy (PDR, subjects with one or more of the following: neovascularization, vitreous/preretinal hemorrhage). Subjects were classified according to changes in the worse eye. Legal blindness was defined as best corrected acuity of 0.1 (6/60) or worse in the better eye. In addition, a pilot study performed in 50 randomly selected adults with type 2 diabetes revealed a Kappa value of 0.73 (95% confidence interval: 0.48-0.98) between observers.

### Assessment of Willingness-to-pay Values

In the present study, WTP was assessed by the following question: "what is the most price (New Taiwan Dollars) that you would be willing to pay for routine screening for DR that reduces the risk of fully blindness?" WTP amounts for a routine screening for DR were elicited by discrete-choice, that is, subjects were presented a single price for a screening program that would yield a specified health change. Subjects either accept or reject the price. By randomly varying the price across a number of different subsamples, the mean WTP could be estimated [11]. To maintain consistency of interview quality, all information on WTP measurements was collected by one well-trained interviewer.

### Data Analysis

In univariate analysis, the independent t-test method or ANOVA was adopted to assess the differences between mean value of WTP. Multiple linear regression was used to assess the independent effects of relevant factors on WTP values after controlling for covariates. Kolmogorov-Smirnov test was used to determine the normal (Gaussian) distribution of refractive errors before linear regression was performed.

The information gathered from study subjects were also evaluated by calculating appropriate standard deviations and 95% confidence intervals (CI). All analyses were performed on SAS^®^ software, version 8.1 (SAS Inc., Cary, NC).

## RESULTS

There were 406 out of 725 eligible participants with type 2 diabetes who attended the survey of WTP evaluation. The overall response rate was 56.0%. Among them, 289 subjects (71.2%) had NDR, 87 subjects (21.4%) had NPDR, 21 (3.0%) had PDR, and 9 (2.2%) were blind. As Table 1 shows, females had higher response rate than male, and participants 60-69 years and 50-59 years responded more frequently than participants in other age groups. When comparing associated demographic characteristics between respondents and non-respondents, most demographic characteristics (e.g., sex, age, duration of type 2 diabetes, and level of education) were similar.

**Table 1.** The response rate of the willingness-to-pay survey among type 2 diabetics in Kinmen.

Variable	Eligible population (n)	Attendant population (n)	Response rate (%)
Sex			
Male	301	156	52
Female	424	250	59

Age (year)			
30-39	6	3	50
40-49	76	39	51
50-59	176	96	55
60-69	233	155	67
70+	234	113	48

Total	725	406	56

Table 2 shows the distribution of WTP values for DR screening among subjects. More than half of the 406 subjects, or 265 (65.3%) said they would be willing to pay for DR screening to reduce blindness. Subjects with no DR had the lowest proportion (59.2%) of willingness-to-pay for DR screening. The average willingness-to-pay value of those answered "YES" (n = 265) was New Taiwan Dollars (NTD) 468.9 ± 327.7 (US dollars 14.3 ± 10.0) (32.8NTD = 1US dollar). Subjects with severe stage DR have higher WTP values for screening than subjects with mild stage DR [NDR (NTD 468.9 ± 327.7) vs NPDR (NTD 450.0 ± 298.8) vs PDR (NTD 683.3 ± 285.5) vs Blindness (NTD 822.2 ± 192.2), F = 13.62, p = 0.0005].

**Table 2.** The distribution of willingness-to-pay of screening for diabetic retinopathy among type 2 diabetics in Kinmen (n=406).

Diabetic retinopathy	Willingness-to-pay		Mean* ± SD (New Taiwan Dollars)

	Yes	No	
	
	n (%)	n (%)	
No diabetic retinopathy (n=289)	171 (59.2)	118 (40.8)	440.1 ± 331.6
Non-proliferative diabetic retinopahty (n=87)	69 (79.3)	18 (20.7)	450.0 ± 298.8
Proliferative diabetic retinopathy (n=21)	16 (76.2)	5 (23.8)	683.3 ± 285.5
Legal blindness (n=9)	9 (100.0)	0	822.2 ± 192.2

Total (n=406)	265 (65.3)	141 (34.7)	468.9 ± 327.7

P-value for ANOVA = 0.0005

* : Only for those with "willing-to-pay = yes"

SD: standard deviation

Table 3 shows the results of univariate analysis of willingness-to-pay values for DR screening among subjects. The education [senior high school or higher (535.4 ± 333.6) vs junior high school or lower (450.0 ± 368.2) vs illiteracy (451.4 ± 321.7), F = 7.99, p = 0.04], number of other chronic diseases [>2 (484.3 ± 331.9) vs 0 or 1 (415.2 ± 318.3), t = 3.42, p = 0.03], and income level [>20,000 NTD per month (513.5 ± 375.1) vs <20,000 NTD per month (459.8 ± 348.6), t = 3.07, p = 0.04] were related to WTP values. In addition, the effects of independent factors on WTP values were also examined by multiple linear regression. Table 4 shows that in addition to education level, degree of DR was an independent factor affecting WTP for DR screening after adjusting for confounders. Age (p = 0.07) and income level (p = 0.08) were borderline significant related to WTP values.

**Table 3.** Univariate analysis of demographic and biochemical variables related to willingness-to-pay of screening for diabetic retinopathy among type 2 diabetics in Kinmen (n=265).

Variable	No (%)	Willingness-to-pay (Mean+SD)*	p-value for t-test or ANOVA
Sex			
Male	108 (40.8)	465.9 ± 319.9	0.86
Female	157 (59.2)	473.1 ± 340.3	

Age (year)			
-59	79 (29.8)	463.7 ± 325.6	0.7
60+	186 (70.2)	481.0 ± 334.4	

Duration of type 2 diabetes (year)			
10-14	245 (92.5)	435.0 ± 243.4	0.63
15+	20 (7.5)	471.6 ± 333.9	

Education			
Senior high school or higher	18 (7.0)	535.4 ± 333.6	0.04
Junior high school or lower	63 (24.5)	450.0 ± 368.2	
Illiteracy	176 (68.5)	451.4 ± 321.7	

Income level (New Taiwan Dollar/month)			
20,000+	59 (22.3)	513.5 ± 375.1	0.03
-19,999	206 (77.7)	459.8 ± 348.6	

Marriage			
Yes	230 (87.8)	464.6 ± 325.6	0.5
No or widow	32 (12.2)	506.3 ± 335.0	

Body mass index (Kg/m^2^)			
-24	149 (56.2)	473.5 ± 330.0	0.8
25+	116 (43.8)	462.9 ± 326.1	

Total cholesterol (mg/dL)			
-199	171 (64.5)	486.0 ± 328.0	0.25
200+	94 (35.5)	437.8 ± 326.7	

Triglyceride (mg/dL)			
-199	173 (65.3)	465.3 ± 324.3	0.81
200+	92 (34.7)	475.5 ± 335.7	

Hemoglobun A1c (%)			
-6.9	139 (52.5)	446.0 ± 316.4	0.28
7.0+	126 (47.5)	489.6 ± 337.5	

Urine protein/Urine creatinine			
-0.19	188 (70.9)	467.8 ± 334.0	0.94
0.20+	77 (39.1)	471.4 ± 314.1	

Medication			
Oral hypoglycemic agents	261 (98.5)	469.9 ± 330.0	0.67
Insulin injection	4 (1.5)	400.0 ± 81.6	

Number of other chronic diseases			
0 or 1	232 (87.5)	415.2 ± 318.3	0.03
2+	33 (12.5)	484.3 ± 331.9	

P-value of Komogorov-Smirnov test of normal distribution for WTP value =0.056.

*: Only for those with "willing-to-pay = yes", New Taiwan Dollar

SD: standard deviation

**Table 4.** Multiple linear regression on the associated factors related to the willingness-to-pay values of screening for diabetic retinopathy among type 2 diabetics in Kinmen.

Variables	*β*	SE	95% CI	p-value
Intercept	586.49	117.92	354.18; 818.79	<0.0001

Sex (female vs male)	10.61	47.10	-82.18; 103.40	0.82

Age (60+ vs -59 yearrs)	41.00	22.87	-86.05; 74.06	0.07

Diabetic retinopathy				
Non-proliferative diabetic retinopathy vs no diabetic retinopathy	34.52	51.14	-66.24; 135.29	0.50
Proliferative diabetic retinopathy vs no diabetic retinopathy	243.99	100.22	46.55; 441.44	0.02
Legal blindness vs no diabetic retinopathy	420.06	112.78	197.88; 642.24	0.0002

Duration of type 2 diabetes (15; vs -14 years)	-34.13	81.99	-195.66; 127.40	0.68

Education				
Junior high school or lower vs illiteracy	81.70	46.62	-9.68; 173.08	0.14
Senior high school or higher vs illiteracy	113.89	53.66	8.72; 219.06	0.04

Income level (20,000+ vs -19,999 new Taiwan dollars/month)	53.77	60.73	-65.27; 172.80	0.09

Marriage (yes vs no+widow)	-54.05	65.55	-183.19; 75.09	0.41

Body mass index (25+ vs -24 Kg/m^2^)	14.50	45.15	-74.45; 103.46	0.75

Total cholesterol (200+ vs -199 mg/dL)	-111.24	55.67	-220.91; 15.74	0.18

Triglyceride (200+ vs -199 mg/dL)	80.23	54.76	-27.66; 188.12	0.14

Hemoglobin A1c (7+ vs -6.9 %)	-73.40	46.41	-164.83; 18.03	0.12

Urine protein/Urine creatinine (0.2+ vs -0.19)	3.30	51.19	-100.55; 101.15	0.99

Medication (insulin injection vs oral hypoglycemic agents)	22.05	163.55	-300.15; 344.25	0.89

Other chronic diseases (2+ vs 0 or 1)	34.28	32.14	-97.60; 69.05	0.29

P-value of Komogorov-Smirnov test of normal distribution for WTP value =0.056

SE: standard error, CI: confidence interval

## DISCUSSION

According to welfare economic theory, the benefit to an individ­ual of a service or intervention is defined as that individual's maximum WTP value for the service or intervention.^3^ The benefit to society of the intervention is the sum of each individual's WTP value.^12^ Evaluation of WTP values as an instrument for patients'satisfaction with DR screening could help explain how much a person values a screening, and whether and how much he/she would pay to get a special screening for DR. However, one inevitable limitation of WTP analysis is that it is entirely hypothetical. Regardless of the subject's response to the instrument, at the conclusion of the interview, the subject had not yet spent any real money. What individuals state they would do and what they actually do may be quite different.^4^

Recent literature on economic evaluation of health and health care has shown increasing interest in the use of WTP questionnaires as a measure of health benefits. Although WTP studies have been performed to assess the relative values of angina treatment,^13^ radiographic contrast dye,^14^ treatment of otitis media,^15^ therapy for lowering intraocular pressure,^4^ and cataract surgery,^16^ they have not been used for ophthalmic screening. In Taiwan, to the best of our knowledge, this study was the first attempt to investigate WTP values in the population with type 2 diabetes. We thought a WTP questionnaire would help determine the most acceptable price of DR screening to adults at risk, that is, those with type 2 diabetes. In the present study, 34.7% of subjects did not want to pay a screening fee for DR, suggesting that they did not believe DR status would limit their quality of life. WTP values were statistically significantly higher in subjects with more advanced DR than those with less advanced DR. This implies that experience of DR associates it with impaired quality of life and thus such subjects would pay to avoid the sequelae of visual impairment. Furthermore, subjects with a higher level of education were more likely to pay for desirable attributes, and to pay greater amounts. More educated individuals may place greater value on convenience and absence of adverse effects, or they may have possessed a better understanding of the effects of DR.^4^

Both WTP and time trade off are useful instrument for global assessment of quality of life. Previous studies also indicate the close association between WTP and time trade off.^17^ Based on the same screening subjects, our previous results found that different degrees of DR in subjects with type 2 diabetes do decrease utility values from the patient's preference-based viewpoint.^18^ This study not only implies the positive correlation between WTP and degree of stage of DR, but also reveals that blind subjects are willing to trade more remaining life years than those not blinded. In addition, it has been suggested that subjects with a higher level of education are more likely to pay more for desirable attributes and more likely to pay greater amounts.^17^ Our results show a similar trend without statistical significance. Because only 18 subjects had education at senior high school or above, the statistical power may be influenced.

There were several strengths of methodology in this study. First, the WTP approach involves valuation of benefits in the same unit as costs, which is required for advising decision makers on efficient allocation,^5^ and offers a better potential for capturing all relevant patient, option and altruistic value benefits for a health intervention. Second, a community-based study is more likely to avoid selection bias. Third, we have adjusted for other possible non-ophthalmologic health related factors which might confound the WTP value of ophthalmic screening by using regression model.

On the other hand, this study had some weaknesses. Because Kinmen is an offshore island a long distance from Taiwan, results may not be reproducible. A low response rate in this study was another drawback. Although the sample size was comparative small, we still had sufficient statistical power to conduct the study (power = 85%, *a* = 0.05).^18^ The small sample size in subjects with PDR or legal blindness might bias the results. In addition, the demographic characteristics such as sex, age and duration of diabetes were similar between responders and non-responders, it implied that diabetics studied may be representative of the non-responders after adjustment for these factors. Nevertheless, we did not have any eye screening for non-responders. The comparison of whether the prevalence of degree of severity similar between two groups is difficult and selection bias might be included. Another drawback is the generalizability of the results. Due to the limited screening time, we could not precisely know the "true" WTP values for DR screening in each subject. Finally, our measurements were conducted at only a single point in time, and, by clear inference, cannot be used to reflect long-term WTP values. Further epidemiological and follow-up investigations with larger study sample sizes are needed to better understand the risk perception and willingness to pay to reduce potential health risks among populations with type 2 diabetes.

In conclusion, we have quantified the WTP values for community-dwelling adults with type 2 diabetes with or without DR. Increased degree of DR increases the WTP in this population, after adjusting for confounders.
